# Dyslexia Polygenic Index and Socio-Economic Status Interaction Effects on Reading Skills in Australia and the United Kingdom

**DOI:** 10.1007/s10519-025-10230-4

**Published:** 2025-09-24

**Authors:** Diana Bicona, Hayley S. Mountford, Elinor C. Bridges, Pierre Fontanillas, Nicholas G. Martin, Simon E. Fisher, Timothy C. Bates, Michelle Luciano

**Affiliations:** 1 Department of Psychology, School of Philosophy, Psychology and Language Sciences, University of Edinburgh, Edinburgh, UK; 2 23andMe, Inc, Sunnyvale, CA USA; 3 Genetic Epidemiology Laboratory, QIMR Berghofer, Brisbane, Australia; 4 Language and Genetics Department, Max Planck Institute for Psycholinguistics, Nijmegen, Netherlands; 5 Donders Institute for Brain, Cognition and Behaviour, Radboud University, Nijmegen, Netherlands

**Keywords:** Gene x environment interaction, Polygenic index, Reading, Spelling, Socio-economic status, School

## Abstract

Literacy is a significant predictor of important life outcomes, such as attained education and income (Ritchie and Bates in Psychol Sci 24(7):1301–1308, 2013. 10.1177/0956797612466268) yet difficulties in reading and spelling are common. Both genetic and environmental factors account for individual differences in reading and spelling abilities (Little et al. in Behav Genet 47:52–76, 2017. 10.1007/s10519-016-9810-6), but there is some evidence that genetic factors can be moderated by environmental factors, many of which relate to differences in socio-economic status (SES). Studies in the US indicate that the heritability of reading and spelling abilities is higher in higher SES environments (Hart et al. in J Child Psychol Psychiatry 54(10):1047–1055, 2013. 10.1111/jcpp.12083; Friend et al. in Psychol Sci 19(11), 2008. 10.1111/j.1467-9280.2008.02213.x). Because countries differ in terms of factors such as education access and social mobility, the genetics (or simply gene) x SES interaction may or may not be present in other populations. Here, we utilise summary statistics from a well-powered genome-wide association study on dyslexia (Doust et al. in Nat Genet 54:1621–1629, 2022. 10.1038/s41588-022-01192-y) to construct polygenic indices in two cohorts of children/adolescents in Australia (N = 1315) and the United Kingdom (N = 5461 at age 7; N = 4306 at age 16), and test whether the effect of measured genes on variation in reading ability is moderated by family SES. While polygenic indices and SES both showed statistically significant effects on reading and spelling performance, no interaction effect was found. These results are contrary to results of some twin studies in the United States that have found an interaction effect. Yet, these findings support the broader literature on gene x SES interaction that mostly report no such interaction in other cognitive traits outside the United States suggesting country differences in how strongly SES relates to education quality.

**Supplementary Information:**

The online version contains supplementary material available at 10.1007/s10519-025-10230-4.

## Introduction

Reading and spelling proficiency is a significant predictor of important life outcomes such as future income (Herrera-Araujo, et al. 2017) and health (DeWalt, et al. 2004), yet people differ in their achieved literacy skills. Individual differences in these abilities are attributed to both genetic and environmental influences (Little et al. 2017), many of which are tied to differences in family socio-economic status (SES). There is some evidence from twin studies that genes interact with the environment to influence variation in reading skills (Hart et al. 2013; Friend et al. 2008). However, no research has examined whether genetically associated predisposition to poor reading (based on individuals’ genotype) interacts with SES. The present study therefore examines the interaction between polygenic indices associated with dyslexia (a strong genetic correlate of quantitative reading skill; Doust et al. 2022) and SES on reading achievement in two population cohorts—Australia and the United Kingdom.

Dyslexia is a specific learning difficulty associated with difficulties in reading, spelling and/or writing abilities that persevere despite appropriate learning opportunities and adequate intelligence (Dyslexia Scotland 2009). It is important to note that although specific definitions of dyslexia differ, they generally converge on these core characteristics with phonological processing difficulties particularly important (Carrol et al. 2025). Estimates of prevalence differ depending on the diagnostic criteria and cut-off point used. Typically, performance 1 or 2 standard deviations below the population mean on reading achievement tests are indicative of dyslexia, resulting in estimated prevalence between 5–12% (e.g., Wagner et al. 2019; Katusic et al. 2001). But dyslexia is often diagnosed with respect to learning in other areas so more general weaknesses in cognitive abilities would not be considered a specific learning difficulty, and the lower extreme of reading ability would partly capture such variation. Genetically, quantitative measures of reading skill are strongly, but not perfectly, correlated with dyslexia (~ 0.75), with the implication being that genes are mostly operating along the continuum (Doust et al. 2022). Considering the high prevalence of dyslexia in the population and the barriers it presents for academic learning, better understanding of risk factors and potential moderators is important.

It is a well-replicated finding that a significant proportion of the variance in reading and spelling abilities can be explained by genetic influences, with these influences becoming more significant and stable with increased age (Little et al. 2017). Heritability estimates from twin studies range from 30 to 80% depending on the age of assessment and sample characteristics (Bates et al. 2004; Tosto et al. 2017; Samuelsson, et al. 2008). Recent meta-analyses of twin studies reveal an average heritability of 57% (Little et al. 2017) for reading comprehension (with no differences in heritability between six countries) and 80% for spelling skill (Andreola, et al. 2021). Genome-Wide Association Studies (GWAS) of reading- and language-related skills indicate that these abilities, as with most cognitive traits, are highly polygenic, meaning they are affected by a large number of single-nucleotide polymorphisms (SNPs), each having a small effect; one significant variant was identified in the most recent scan (Luciano et al. 2013; Eising et al. 2022). A well-powered GWAS of dyslexia identified 42 independent genome-wide significant loci; and polygenic indices for dyslexia constructed in independent cohorts explained up to 6% of variance in quantitative reading and spelling abilities (Doust et al. 2022).

## Gene x SES Interaction

The Scarr-Rowe effect (Scarr-Salapatek 1971; Rowe et al. 1999) predicts that heritability of cognitive traits is higher in high SES environments than in low SES environments. The rationale is that children growing up in high SES families have more opportunities to attain their genetically associated potential, whereas environmental disadvantages will limit opportunities to do so for children growing up in low SES families. Consequently, genetic influences will not be as significant a source for individual differences in low SES environments (Scarr-Salapatek 1971). While Eaves and Jinks (1972) were unable to replicate the findings of the Scarr-Salapatek (1971) study when employing a more rigorous statistical analysis, a large and well-powered meta-analysis by Tucker-Drob and Bates (2016) provides evidence supporting the existence of gene-SES interaction effects on academic achievement and intelligence in the United States (US). For reading ability, effects of SES are associated with differences in specific environments that promote development of reading, such as number of books at home, parental involvement and quality of teaching (Aikens and Barbarin 2008). However, it is worthy to note that these environments are not random and contain substantial genetic effects themselves (e.g., Hart et al. 2021), complicating the causal interpretation of any such findings.

Support for gene x SES interaction effects on reading and spelling abilities comes from several twin studies from the US that have focussed on low and high extremes of achievement. For reading difficulties (defined as performance more than 1.5 SDs below sample mean) measured in children, heritability was higher and shared environmental influence lower when their parents had higher education, and more equal contributions from genes and shared environment were observed when their parents had lower education (Friend et al. 2008). In contrast, heritability for high reading ability (defined as performance 1 SD above the sample mean) was found to be higher for children whose parents had lower versus higher education (Friend et al. 2009), this interaction being present for reading ability in kindergarten (mean age 6.3 years), first grade (mean age 7.5 years), and second grade (mean age 8.4 years). However, the effect was only replicated for sixth grade (mean age 11.5 years) and not first grade (mean age 7.1 years) in a nationally representative twin sample from the United Kingdom (UK; Friend, et al. 2009). The authors proposed that deviations in children’s reading performance from environmental expectations— such as poor outcomes in more favourable literacy environments (high SES) or exceptional outcomes in less supportive contexts (low SES)—indicate a heightened influence of genetic factors, as opposed to situations where reading performance aligns with environmental predictions. But some US studies have failed to replicate gene x SES effects. For instance, when investigating reading achievement (both disability and full range reading variation) and parental educational attainment (EA) in the Minnesota Twin Study (n = 3779) and in the Sibling Interaction and Behaviour Study, a large adoption study of sibling pairs (n = 1232), no interaction effect was found (Kirkpatrick et al. 2011). Potential explanations for such discrepancies within the same population might include cohort effects and differences in reading achievement measurement.

Gene x SES interaction effects on reading ability have also been studied in Australia across several timepoints in childhood and adolescence (Grasby et al. 2019). The shared environment influence on reading ability was higher for Grade 3 children (age 8–9 years) in families where parents had low EA compared to high EA families, but this moderation effect disappeared when the same pupils were tested at Grade 5 (age 10–11) and Grade 7 (age 12–13). Overall, evidence from twin studies is inconsistent, with gene x SES interaction effects on reading achievement having some support in the US, but little support in the UK and Australia. Because SES is a significant predictor of educational outcomes in all the Organization for Economic Cooperation and Development (OECD) member countries (OECD 2010), further studies to examine gene x SES interaction are necessary.

Previous gene x SES interaction studies in the area of reading skill have used a twin design, but polygenic indices allow us to examine genetic effects directly using genotype data. Polygenic indices are a composite value representing the combined effect of many SNP effects estimated for a trait, typically from a well-powered GWAS. They have successfully been used to predict phenotypic variation in a range of cognitive traits (Harden et al. 2020; Selzam et al. 2017a, b). For instance, the polygenic index for years of education explained up to 5% of the variance in reading performance between the ages of 7 and 14 (Selzam et al. 2017a) and up to 9% of the variance in educational achievement at age 16 (Selzam et al. 2017b) in the TEDS cohort in the UK. Harden et al. (2020) used polygenic indices to examine gene x SES interaction in maths tracking in the US, finding that individuals with higher educational attainment polygenic indices were less likely to be enrolled into advanced maths classes if attending low SES schools. Similarly, attending a high SES school was associated with a buffering effect for those with lower educational attainment polygenic indices.

Following from the Doust et al. (2022) GWAS on dyslexia, it is now possible to investigate whether SES can modify the effect of polygenic indices associated with dyslexia, the objective of this study. While a GWAS for reading skill itself would be ideal, the largest GWAS of word reading accuracy is in a modest sample of ~ 33 K (Eising et al. 2022) in contrast to the dyslexia GWAS in over 1 million participants. In this study, we compare results from adolescents in Australia and the UK and further examine children (aged 7 years) in the UK sample. School and family-level SES measures are considered as potential environmental moderators of the effect of a dyslexia polygenic index on observed reading performance.

## Methods

### Australian Cohort—Brisbane Adolescent Twin Study

The Australian cohort consisted of 1315 Australian adolescents (mean age 17.6 years, SD = 3.2, 53% females) from 604 families from the Brisbane Memory, Attention and Problem Solving (MAPS) study, the 16-year-old wave of the Brisbane Adolescent Twin Study (BATS; Wright and Martin 2004). This included only one monozygotic twin from a pair, selected at random. DNA was extracted from blood samples (> 90% participants) or buccal cells (Wright and Martin 2004). Genetic data were imputed using 1000 Genomes Phase 3 (v.20101123). Further information on quality control can be found in Doust et al. (2022) and Luciano et al. (2013).

### Reading and Spelling Measures

Reading was assessed using the Components of Reading Examination (CORE; Bates et al. 2004), a 120-word extended version of the Castles and Coltheart (1993) test to increase complexity for a sample of older adolescents. These 120 words included 40 regular words (e.g., *tail*), 40 irregular words (e.g., *meringue)* and 40 nonwords (e.g., *phleptish*), presented in a random order. Regular and irregular word spelling were assessed by verbally presenting 18 words from each CORE test. Nonword spelling was assessed by asking participants to regularise spellings of irregular words. There were thus six available measures: regular, irregular and nonword reading, and regular, irregular and nonword spelling. More information and exact materials are available in Bates et al. (2004).

### Parental SES

SES index was calculated individually for each parent, derived from the information on their occupation and level of education (Bates et al. 2018). When information on their occupation was available, socio-economic status was directly assigned based on Australian Socioeconomic Index 2006 (AUSEI06) occupational status scale (McMillan et al. 2009). This scale was developed by the Australian Bureau of Statistics, based on the data collected in the 2006 Australian Census. The values range from 0 (lowest) to 100 (highest), and are imputed based on an individual’s current occupation. When information on occupation is not available or for individuals not in paid unemployment, AUSEI06 is assigned based on their educational attainment (McMillan et al. 2009). A single measure of parental SES was used – the average SES index per family.

### School Type

Participants were asked to provide information on which school they were attending. This information was available for 586 participants. These schools were coded as State or Private based on publicly available from The Queensland Schools Directory dataset (Queensland Government 2022). Private schools included both Catholic and independent schools and are fee-paying schools, another marker of SES. Additionally, attending a private school in Australia is associated with more positive student outcomes, such as higher university entrance scores (Ryan and Watson 2010), so might indicate a more literacy rich environment. Although these effects are less robust for Catholic schools, a decision was made to merge independent and Catholic schools in a joint ‘Private school’ category due to the small subsample of participants for whom school information was available.

### UK Cohort: 1958 National Child Development Study (NCDS)

NCDS is an ongoing birth cohort study that follows the lives of more than 17,000 people in the UK born in a single week in 1958 (National Child Development Study, n.d.). A DNA sample was collected at the 45-year follow up for a subsample of 6431 individuals (Power and Elliott 2005). After excluding individuals who did not pass quality control checks, the sample decreased to 6410, genotyped on seven different arrays (Bridges et al. 2023). Genetic data imputed using the Haplotype Reference Consortium were used, details of which can be found in Bridges et al. (2023). For this study, phenotypic data were used from the Age 7 years sweep and Age 16 years sweep.

### Reading Measures at Age 7

The Southgate Group Reading Test, a 30-item test of word recognition and comprehension (Shepherd 2012) was administered to children at school by a teacher. For 16 questions, children were given a picture of an object and had to circle the correct word describing the object (Southgate 1962). For remaining questions, the teacher read out the word and the child had to circle the correct answer. This test was particularly suited to identify poor readers (Shepherd 2012) showing a negatively skewed distribution of test scores. The child’s main teacher rated their reading ability as compared to other children of the same age, producing an ordinal scaled variable, where the lowest value (1) was assigned to “Non-reader or recognises very few words” and highest value (5) was assigned to “Avid reader or reads fluently and widely in relation to his age”. The teacher also rated the level that the child had reached in a reading scheme. Option *“Don’t know or inapplicable”* (n = 66) was removed to create an ordinal scale. The lowest value (1) was assigned to *“On pre-reading activities only”*, whereas the highest value (6) was assigned to *“Beyond basic reading scheme”.*

### Reading Measures at Age 16

There were 5 different reading measures available at this timepoint. However, two measures (self-assessed English ability and self-reported reading frequency) did not correlate with other reading variables in this and other NCDS sweeps (Bridges et al. 2023), and thus were not included in composite score calculation. Another measure captured whether the child reads well enough to cope with everyday needs, as assessed by the child’s main teacher. However, there was an extremely small number of participants (n = 40) considered unable to read well enough to cope, making it unsuitable for statistical analysis. As recommended in Bridges et al. (2023), a reading skill composite score was created based on the following variables: (1) a 35-item reading comprehension test, based on the Watts-Vernon test of reading ability and designed specifically for the NCDS (Shepherd 2012). For each question, the child had to choose from 5 words that completed sentences. (2) The child’s main teacher assessed their academic aptitude for English. The options included “*Capable of obtaining an A-level or Higher-grade pass in this subject”*; *“Above average. Capable of obtaining O-level or O-grade or CSE grade one”*; *“Of average ability in this subject. Capable of obtaining a CSE pass, grades 2–4”*; *“Below average. A possible CSE entrant”*; *“Little, if any, ability in this subject”*; *“Don’t know”*. *“Don’t know*” (n = 17) was removed to transform this variable into a scale of 5, where the highest value was assigned to children believed capable of obtaining the highest grade.

### Parental SES

A single SES measure was used for the analysis: father’s occupation when the child was aged 7 years as reported by the child’s mother. Father’s occupation is an appropriate measure of SES for the NCDS sample who were born in 1958 when fathers were the primary breadwinner; in the UK, it captured education, income and social prestige associated with a specific occupation (Bland 1979). Father was specified as the male head of the household, and hence was not limited to biological fathers. Valid answers were coded by NCDS researchers in 8 categories as follows, in accordance with General Register Office (1960) classification, further distinguishing between manual and non-manual occupations: 1. No male head of the household, 2. Class I: professional, 3. Class II: intermediate, 4. Class III: other non-manual, 5. Class III: skilled manual, 6. Class IV: partly skilled non-manual, 7. Class IV: partly skilled manual, and 8. Class V: unskilled. A rank of 1–8 was created, where the highest value was assigned to Class 1, and the lowest rank to single mothers. As noted by other studies using the NCDS dataset (Lacey et al. 2011), single motherhood at this time was an indicator of socio-economic disadvantage. Whereas other studies using NCDS data (e.g., Lacey et al. 2011) have typically added such cases to the lowest SES category (unskilled), this study left it as a category on its own, assigning it the lowest rank. This is because single parenthood is associated with poorer outcomes, even after controlling for other SES indicators (OECD 2010).

### Dyslexia Polygenic Indices

Polygenic indices were constructed using the GWAS summary statistics (without genomic correction) of Doust et al. (2022) who analysed self-reported dyslexia diagnosis from a 23andMe, Inc. online survey of their consenting research participants. Participants were of European ancestry and included 51,800 dyslexia cases and 1,087,070 controls. Polygenic indices were calculated in the Australian and UK cohorts using PRSice-2 software, in accordance with protocol detailed in Choi and O’Reilly (2019). We used polygenic indices constructed from all SNPs as they were shown to explain the most variance in the reading and spelling variables in Doust et al. (2022). All polygenic indices were standardised prior to the analysis, with higher values relating to increased predisposition to dyslexia.

## Statistical Analyses

Internal reliabilities of the reading and spelling measures were assessed using Cronbach’s alpha, calculated with the *alpha* and *omega* functions from the psych package (Revelle, 2024). Principal component analysis (PCA) was performed on the strongly correlated reading and spelling measures within cohorts to extract a general reading ability factor explaining the most variance (i.e., the first unrotated principal component). The *principal* function from the *psych* package (Ravelle 2024) in R (version 4.4.1) was used and applied to the correlation matrix (Jolliffe and Cadima 2016). In the Australian cohort, all six variables demonstrated excellent internal consistency (Cronbach’s α = 0.90). McDonald’s omega total and omega hierarchical were both 0.92, indicating high reliability of the composite score. This component score was then residualised on age, age squared, and sex. This was done using the lm function from the stats package in R, and the resulting residuals were standardized. In the UK cohort, at age 7, all variables contributed positively to the scale with overall Cronbach’s alpha ranging between 0.81–0.83, indicating good internal consistency. Excellent reliability of the composite score was indicated by Mcdonald’s omega total and omega hierarchical of 0.88. At age 16 both variables demonstrated good internal consistency (Cronbach’s α = 0.72) with a McDonald’s omega total and omega hierarchical estimated at 0.83, indicating good reliability of the composite score. The reading component scores were adjusted for sex and standardised. Distributions of adjusted scores were visually inspected for outliers for both samples. Scores lower than 4 standard deviations below the mean were removed in the Australian sample. No extreme data points were observed in the UK sample.

Interaction effects were modelled by linear regression, but in the Australian cohort, linear mixed effects models were used to account for family (i.e., sibling) relationships within the sample (Dunn and Smyth 2018). Models were fitted using the *lme* function from the *nlme* package (Pinheiro et al. 2024). We constructed four different models. First, the standardised reading and spelling component was regressed on the standardised polygenic index (plus 20 ancestry principal components and imputation run—a variable indicating the specific genotype imputation batch) with family relationship modelled as a random effect (Model 1). Model 2 included standardised parental socio-economic status as an additional fixed effect, alongside the interaction term of the polygenic index and parental socio-economic status. In Model 3, school type was instead examined as a moderator of the polygenic index for individuals’ reading and spelling performance. Finally, to examine whether school effects are independent of parental SES, the effects of school type were assessed alongside parental SES and polygenic indices (Model 4). For the UK cohort, linear regression models were fitted using the *lm* function from the *stats* package (R Core Team 2024). Model 1 tested the effect of dyslexia polygenic indices (and including 10 ancestry principal components and genotyping array), to assess explanatory power of polygenic indices. Model 2 tested the effects of socio-economic status alongside polygenic indices, as well as their interaction. All effects were considered as statistically significant at α < 0.05. Each regression model was checked for linearity, normality of residuals, homogeneity of variance, outliers, and high leverage points.

Statistical power for detecting interaction effects was assessed post-hoc for each sample, using simulation-based approach, where 1000 datasets were generated using the study’s design and the potential effect size, and the proportion of models detecting a significant effect (*p* < 0.05) was calculated to estimate power. Analysis code for each cohort can be found in supplementary material.

## Results

### Australian Cohort

After excluding one monozygotic twin from each pair, the analytic sample size was 1640, reducing further to 1307 for analysis including SES. Parallel analysis and scree plot suggested keeping one component which accounted for 71% of the variance among the reading and spelling variables. The highest loadings (Supplementary Table 1) were for irregular and nonword reading (0.91), followed by regular word spelling (0.86), regular word reading (0.84), irregular word spelling (0.83) and nonword spelling (0.69). Visual inspection of the distribution of the standardised scores suggested to remove scores lower than 4 standard deviations below the sample mean (n = 8).

The distribution of polygenic indices across different SES levels showed that, on average, those in the lowest SES tier had the highest dyslexia polygenic indices, suggestive of gene-environment correlation (see Table 1). Model 1 showed that a one standard deviation increase in the dyslexia polygenic index was associated with a 0.2 standard deviation decrease in reading and spelling performance (*p* < 0.001), explaining 4.4% of variance (95% CIs 3.7–7.7%) in general reading and spelling performance (Supplementary Table 2). In Model 2, SES showed an independent effect on the reading and spelling composite score beyond the polygenic index, increasing model fit to 10% (95% CIs 8.4–14.5%) (see Table 2). One standard deviation increase in SES was associated with 0.22 standard deviation increase in reading and spelling performance (*p* < 0.001). However, there was no significant interaction effect between SES and dyslexia polygenic index (*p* = 0.29). But given the observed interaction effect size of 0.03, there was low statistical power (17%) to detect this.

**Table 1:** Average dyslexia PGI in low, average, and high groups of SES in the Australian cohort

SES	Average Polygenic Index	SD	N
Low (0–30)	0.16	0.99	193
Average (31–70)	− 0.04	0.94	809
High (71–100)	− 0.01	0.92	313

On the continuous scale, the average socioeconomic index was 51.5 (SD = 24.6)

**Table 2:** Dyslexia PGI x SES interaction model estimates for the Australian Cohort (Model 2)

Predictor	Value	Std. Error	DF	t-value	*p*-value
(Intercept)	0.05	0.03	553	1.42	0.15
Dyslexia PGI	− 0.20	0.03	1260	− 7.11	< 0.01
SES	0.22	0.03	556	6.84	< 0.01
Dyslexia PGI ~ SES	0.03	0.03	1266	1.06	0.29

In Model 3 (see Table 3), attending a state school instead of private school was associated with a 0.22 standard deviation decrease in reading and spelling performance (*p* < 0.05). School type did not moderate the association between dyslexia polygenic index and reading and spelling score (*p* = 0.25). When including parental SES as a predictor (Model 4), the effect of school type reduced by almost half and was no longer significant (see Table 3), suggesting that school effects are largely accounted for by differences in family-level SES. However, this subsample was smaller (n = 405) and the main effect of state school (a binary measure) was estimated with greater error. This model explained 9.7% (95% CIs 9.1–20.1%) of variance in the reading and spelling composite score. Supplementary Table 2 shows all models in a single table for ease of comparison.

**Table 3:** Dyslexia PGI x school type interaction model estimates (Model 3) for the Australian sample

Predictor	Value	Std. Error	DF	t-value	*p*-value
*Model 3*					
(Intercept)	0.20	0.08	214	2.54	< 0.01
Dyslexia PGI	− 0.23	0.08	380	− 2.73	< 0.01
State School	− 0.22	0.11	247	− 1.93	0.05
Dyslexia PGI ~ state school	0.13	0.11	379	1.14	0.25
*Model 4*					
(Intercept)	0.14	0.08	216	1.68	0.09
Dyslexia PGI	− 0.22	0.08	379	− 2.75	< 0.01
State School	− 0.11	0.12	247	− 0.99	0.32
Family SES	0.20	0.06	193	3.22	< 0.01
Dyslexia PGI ~ state school	0.12	0.11	378	1.11	0.27

Model 4 includes an adjustment for family SES

### UK Cohort

## Age 7 Years

Genotype and reading phenotype data were available for 5712 participants but due to missing SES data, the sample reduced to 5461 for the interaction effect model. All three reading measures were highly correlated. Scree plot and parallel analysis suggested to keep one component. This component accounted for 80% of the variance in the available reading measures. Loadings of 0.9 were observed for Southgate Reading Test and Progress in Reading Scheme, and 0.89 for teacher’s ratings.

Average polygenic indices within each SES tier showed that, with an exception for those of single mothers, children at lower SES categories had slightly higher dyslexia polygenic indices (Table 4), consistent with findings for the Australian sample and in line with the presence of gene-environment correlation.

**Table 4:** Average dyslexia polygenic index across SES categories in the UK cohort

SES	Average polygenic index	SD	N
Single mothers	− 0.05	1.06	95
Class V (lowest)	0.06	1.00	221
Class IV manual	0.04	1.02	637
Class IV non-manual	0.03	0.99	72
Class III other non-manual	0.00	1.02	1910
Class III skilled manual	− 0.04	0.96	427
Class II	-0.03	0.99	668
Class I (highest)	-0.05	0.96	276

In the regression model (see Table 5), a one standard deviation increase in dyslexia polygenic index was associated with a 0.11 standard deviation decrease in the reading component score (*p* < 0.001), explaining 1.6% (95% CIs 1.2–2.4%) of its variance. Adding SES to the model increased model fit to 8% (95% CIs 7–9.8%). A one standard deviation increase in SES was associated with a 0.26 standard deviation increase in reading component score (*p* < 0.001). There was no significant interaction effect between dyslexia polygenic index and SES on the reading component score (*p* = 0.16). A post-hoc statistical power analysis indicated a power of 30% to detect the observed interaction effect size of 0.02.

**Table 5:** Dyslexia PGI x SES interaction model estimates for the UK (NCDS Age 7 Sweep) cohort

Predictor	Value	Std. Error	t-value	*p*-value
(Intercept)	0.01	0.01	0.31	0.76
Dyslexia PGI	− 0.11	0.01	− 8.85	< 0.01
SES	0.26	0.01	19.76	< 0.01
Dyslexia PGI ~ SES	0.02	0.01	1.42	0.16

## Age 16 years

Genotype and reading phenotype data were available for 4809 participants but due to missing SES data, the sample reduced to 4306 for the interaction effect model. Following inspection of the correlation matrix and distribution of available measures, PCA for this timepoint was conducted using the reading comprehension test and teacher’s English rating, in line with recommendations in Bridges et al. (2023). The principal component accounted for 86% of variance with equal loadings of 0.93.

The regression model showed that a one standard deviation increase in dyslexia polygenic index was associated with a 0.09 standard deviation decrease in the reading skill composite score (*p* < 0.001), accounting for 1.6% (95% CIs 1–2.6%) of variance in the reading skill composite score (Table 6). Adding SES to the model improved model fit to 12.2% (95% CIs 10.5–14.3%). A one standard deviation increase in SES was associated with a 0.33 standard deviation increase in reading component score (*p* < 0.001). As in the Age 7 sweep, there was no interaction effect between dyslexia polygenic index and SES on reading component scores (*p* = 0.19). A post-hoc statistical power analysis indicated a power of 28% to detect the observed interaction effect size of 0.02.

**Table 6:** Dyslexia PGI x SES interaction model estimates for the UK (NCDS Age 16 sweep) cohort

Predictor	Value	Std. Error	t-value	*p*-value
(Intercept)	0.00	0.02	0.42	0.67
Dyslexia PGI	− 0.09	0.01	− 6.16	< 0.01
SES	0.33	0.01	22.78	< 0.01
Dyslexia PGI ~ SES	0.02	0.01	1.32	0.19

## Discussion

In Australian and UK samples of children and adolescents who were born in different generations, both dyslexia polygenic index and family SES had independent statistically significant effects on reading skill but did not interact to influence achievement. Although post-hoc statistical power estimates indicated that the studies lacked statistical power to detect interaction effects of the observed magnitude (0.03 in Australia and 0.02 in the UK), the small size of the effect even if detectable with improved power suggests it is of little practical import. Together, polygenic indices and socio-economic status explained between 8 and 11.6% variance in reading skill differences between individuals at different ages, generations, and countries. The interaction of genes and state versus private school education was also non-significant, and the significant main effect of school type was explained by family SES. Taken together, the evidence from these two independent cohorts do not support the hypothesis that expression of genetic predisposition to dyslexia is moderated by SES, at least in developed countries with relatively good quality public education systems.

The lack of a polygenic x SES interaction effect is in line with findings of Grasby et al. (2019) who did not find any gene x SES interaction effect on reading ability in a different sample of Australian twins that focussed on total genetic variance. Our study indicates that common genetic variation (as measured by polygenic indices) does not significantly interact with SES to affect reading skill in Australia (in the early 2000s) or the UK (in the 1960/70 s). These results are contrary to studies in the United States that have reported gene x SES interaction for the extremes of reading ability (e.g., Friend et al. 2008, 2009) but are in line with broader literature on gene x SES interaction for other cognitive traits. For instance, in a large, well-powered meta-analysis of 10,000 twin pairs, a gene-SES interaction effect size of 0.074 was found for general intelligence (a moderate correlate of reading achievement) in the US cohort, but not in the cohorts outside the US (e.g., Tucker-Drob and Bates 2016). Interestingly, geographic location within country was differentially related to the heritability of reading comprehension and the strength of gene x SES effects (Shero et al. 2024). In a large Florida twin cohort (n = 2135), the moderating effects observed in the full sample were not generalisable across all geographic sub-samples, likely due to regional variation in income heterogeneity (Shero et al. 2024). This highlights the importance of using geographically sensitive study designs due to role of local environmental contexts such as school quality, community resources, and socioeconomic diversity.

In the Australian sample, attending a state school was marginally associated with a decrease in reading and spelling scores but this was explained by parental SES, indicating that attendance of private or state school largely reflects family-level SES (i.e., access to specific school type) and not differences between the quality of resources or teaching, for instance, between private and state schools. There may be a smaller positive independent effect of private schooling because it had a similar effect size to the polygenic index but with larger standard errors given its binary categorisation. Further, it may be that more fine-grained indicators of the school environment such as teacher experience, class size and school leadership (National Center for Education Statistics 2000) or school literacy environment (e.g., phonics instruction, presence of a library) are important moderating variables of the polygenic effect.

We note that the variance in reading performance explained by polygenic indices (which sample common genetic variants that likely tag rarer causal variants) is still much lower than heritability estimates for reading skill from twin studies. Therefore, it is possible that other genetic effects (including copy number variants and rare genetic variants) or genetic effects that converge in specific biological pathways interact with aspects of the environment. Further, polygenic indices in general are more likely to capture genetic variants that have a similar effect across populations and environments, whereas other genetic variants with higher penetrance might be more sensitive to moderation by environmental influences (de Zeeuw et al. 2019). Also of note is that we used a polygenic indicator of dyslexia whereas our outcomes were continuous measures of reading ability, an imperfect proxy of dyslexia. An equally powerful GWAS on quantitative reading skill would likely improve polygenic prediction but does not yet exist.

Whereas we did not observe a significant gene x environment interaction in either cohort in our study, it is worth highlighting that children growing up in low SES families had higher average dyslexia polygenic indices than children growing up in high SES families, and this was true for both cohorts. However, children of single mothers in the UK did not show a higher dyslexia polygenic risk which may indicate that the group was higher in parental educational attainment despite their broad SES being assumed to be lower. This is consistent with observations for this period that divorce was more common among the more highly educated (Goode 1993). The differences in polygenic indices between SES groups suggests that children are not randomly born into SES environments, with family SES potentially reflecting parental genetic factors (e.g., Bates et al. 2018). Using a design that included genetic data from both parents and offspring, Bates et al. (2018) found that parents’ genetic factors affect educational outcomes of their children via SES, even when these genetic factors are not transmitted to the child. This might partially explain the lower reading performance observed in lower SES groups in our study and others (e.g., Friend et al. 2008; Kieffer 2010). Our results then do not suggest that specific SES environments exacerbate or buffer against genetic predisposition to poor reading, but they support an increased need for literacy support in lower SES families.

## Strengths and Limitations

Some limitations of the dyslexia GWAS should be noted as they are directly relevant to this study as well. Despite the large sample size, the GWAS sample may not be representative of the general population. 23andMe is a consumer genetics and research company, and hence research participants included in this study are a self-selecting group. Additionally, dyslexia prevalence in their sample was 5%, which is at the lower range of dyslexia estimates of 5–10% of school age children (Pennington and Bishop 2009). This will limit the genetic variation that could be detected and therefore the predictiveness of the polygenic index.

There are some specific limitations of the UK cohort used in this study. First, the genotyped subsample of NCDS is potentially lacking representativeness because it contains fewer poor readers than the full sample (Bridges et al. 2023). Another limitation is lack of data on the date of testing. This could be especially impactful for the Age 7 measures as additional months or weeks of schooling could have a substantial effect on children’s reading performance at the time of testing. This may partially explain why polygenic indices explained less variance in reading scores in the UK compared to the Australian cohort; variation related to developmental lags at age 7 will not be present at age 16 and we would not expect these to relate to a dyslexia PGI. Additionally, educational attainment polygenic effects on reading are found to be weaker at earlier ages (Selzam, et al. 2017a, b). For the Age 16 sweep in the UK cohort, the variance in reading skill scores explained by polygenic indices alone was even lower. This is potentially due to the nature of the reading measures used in constructing the PC at this age. Reading comprehension taps cognitive processes beyond word recognition/decoding (the core indicators of dyslexia) and the teacher rating of child’s academic aptitude in English could reflect factors beyond reading, such as creative writing.

## Implications and Future Directions

Overall, our study shows that SES does not affect expression of polygenic predisposition to dyslexia in the United Kingdom and Australia. But one must appreciate that the UK results are based on a cohort born in 1958, and since then there has been a decline in daily reading and reading for pleasure outside of school (National Literacy Trust 2025) alongside a rise in income inequality as indicated by the Gini index (World Bank 2023). Similarly, reading attainment has decreased over the last two decades in Australia (Curtis and Nielsen 2025) alongside changes for both countries in educational practices (e.g., greater support for additional learning needs). Such changes might alter the patterns of gene x SES interaction effects across generations so studies on more contemporary data are needed. We found that both genetic and family socio-economic factors independently influence reading achievement, with SES environmental effects being larger. However, these findings are limited by the predictive power of the polygenic index for dyslexia. As GWAS get larger more genetic variance in dyslexia (and quantitative reading skill) will be explained, which might increase the power to detect gene x SES interaction effects in future but these effects are likely to remain small unless future studies also sample more heterogenous populations where the environmental variation is larger. More importantly, future studies which focus specifically on the literacy environment rather than a broad family SES environment are needed to conclude that environmental factors do not interact with genes to influence literacy skills and to further understand what is driving the broader SES main effect. These more specific aspects of the environment might show less genetic confounding and as such could aid the design and implementation of educational policies and reading instruction practice to overcome barriers in the development of proficient reading and spelling skills.

## Supplementary Information

Below is the link to the electronic supplementary material.

Supplementary file1 (TXT 12 KB)

Supplementary file2 (TXT 14 KB)

Supplementary file3 (DOCX 20 KB)

## Data Availability

The full GWAS summary statistics for the 23andMe, Inc. dyslexia study are available through 23andMe to qualified researchers under an agreement with 23andMe that protects the privacy of the 23andMe participants. Datasets will be made available at no cost for academic use. Please visit https://research.23andme.com/collaborate/#dataset-access/ for more information and to apply to access the data. The full NCDS phenotypic dataset is openly available to researchers at the UK Data Service. The genotyped subsample and matched phenotypic data are available from the Centre for Longitudinal Studies at University College London, upon submission of a successful research proposal. The Brisbane Adolescent Twin Study data are not publicly available and would require agreements with the QIMR Berghofer Medical Research Institute for access.
