# Supplementary material for: Dyslexia Polygenic Index and Socio-Economic Status Interaction Effects on Reading Skills in Australia and the United Kingdom
Source: Behav Genet. 2025 Sep 24;55(5):395–406. doi: 10.1007/s10519-025-10230-4 (PMC12494669; doi:10.1007/s10519-025-10230-4)
Supplement: Supplementary file 3 — Supplementary file3 (DOCX 20 KB) [file 10519_2025_10230_MOESM3_ESM.docx]

**Supplementary Tables**

*Table 1. PCA loadings for Brisbane Sample*

| Loadings | PC1 | PC2 |
| --- | --- | --- |
| Irregular word Reading | 0.91 | -0.15 |
| Regular word reading | 0.81 | -0.15 |
| Nonword reading | 0.91 |  |
| Irregular word spelling | 0.84 | -0.18 |
| Regular Word Spelling | 0.86 |  |
| Nonword Spelling | 0.69 | 0.72 |
| Proportion of Variance | 0.71 | 0.1 |

*Table 2. Model Comparison Table for Brisbane Sample*

|  | Model 1 | Model 2 | Model 3 | Model 4 |
| --- | --- | --- | --- | --- |
| Intercept | 0.01 | 0.05 | 0.20 | 0.14 |
| Polygenic Index (PGI) | **-0.19***** | **-0.20***** | **-0.23*** | **-0.22*** |
| Family SES |  | **0.22***** |  | **0.20**** |
| State School |  |  | **-0.22*** | -0.11 |
| Family SES-PGI interaction |  | 0.03 |  |  |
| School Type-PGI interaction |  |  | 0.13 | 0.12 |
| R-Squared | 0.044 | 0.10 | 0.065 | 0.097 |
| Number of Observations | 1640 | 1307 | 404 | 404 |

*Significance levels indicated by * p < 0.05, ** p < 0.01, *** p < 0.001*

*Table 3. PCA loadings for NCDS Age 7 Sample*

| Loadings | PC1 | PC2 |
| --- | --- | --- |
| Southgate Reading Test | 0.9 | -0.17 |
| Reading ability (teacher’s rating) | 0.89 | -0.28 |
| Level on a reading scheme | 0.89 | 0.46 |
| Proportion of Variance | 0.8 | 0.1 |

*Table 4. PCA loadings for NCDS Age 16 Sample*

| Loadings | PC1 | PC2 |
| --- | --- | --- |
| Watts-Vernon Test of Reading Ability | 0.93 | -0.38 |
| Academic aptitude for English | 0.93 | 0.38 |
| Proportion of Variance | 0.86 | 0.14 |

*Table 5. Model Comparison Table for NCDS Age 7 Sample*

|  | Model 1 | Model 2 |
| --- | --- | --- |
| Intercept | 0.01 | 0.01 |
| Polygenic Index (PGI) | **-0.12***** | **-0.11***** |
| Family SES  Family SES – PGI interaction |  | **0.26*****  0.02 |
| R-Squared | 0.02 | 0.08 |
| Number of Observations | 5712 | 5461 |

*Significance levels indicated by * p < 0.05, ** p < 0.01, *** p < 0.001*

*Table 6. Model Comparison Table for NCDS Age 16 Sample*

|  | Model 1 | Model 2 |
| --- | --- | --- |
| Intercept | 0.02 | 0.01 |
| Polygenic Index (PGI) | **-0.10***** | **-0.09***** |
| Family SES  Family SES – PGI interaction |  | **0.33*****  0.02 |
| R-Squared | 0.02 | 0.12 |
| Number of Observations | 4809 | 4306 |

*Significance levels indicated by * p < 0.05, ** p < 0.01, *** p < 0.001*
